# Chronic Non-infectious Osteomyelitis Mimicking Scurvy as the Presenting Sign of Crohn’s Disease: Case Report

**DOI:** 10.3389/fped.2022.871993

**Published:** 2022-04-13

**Authors:** Alexa Goldfarb, Stefan Breitling, Owen Amadasun, Bridget Kiernan, Gabrielle Gold-von Simson, Selin T. Sagalowsky

**Affiliations:** ^1^Department of Pediatrics, New York University Langone Health, New York, NY, United States; ^2^Department of Pediatrics, New York University Grossman School of Medicine, New York, NY, United States

**Keywords:** inflammatory bowel disease, nutrition, autism spectrum disorder (ASD), osteomyelitis, case report

## Abstract

Chronic non-infectious osteomyelitis (CNO) is a rare, inflammatory process associated with pediatric inflammatory bowel disease (IBD). Signs and symptoms of CNO parallel scurvy, a nutritional deficiency that can affect children with autism spectrum disorder (ASD). This is the first report of a child initially thought to have scurvy, then subsequently diagnosed with CNO as the presenting manifestation of Crohn’s disease. This case enhances the literature elucidating extra-intestinal manifestations of IBD and pediatric nutritional deficiencies.

## Introduction

Chronic non-infectious osteomyelitis (CNO) is an elusive, inflammatory process that can present with bony pain and is associated with pediatric inflammatory bowel disease (IBD). Signs and symptoms of CNO can mimic scurvy, a rare nutritional deficiency known to affect children with autism spectrum disorder (ASD). This case of a young child with ASD takes us on a journey through the differential diagnoses of limb pain in children, pediatric nutritional deficiencies, and bone marrow malignancies, ultimately elucidating one of the rare extra-intestinal manifestations of IBD.

## Case Presentation

A 5 year-old male with autism spectrum disorder (ASD) presented to the pediatric emergency department (ED) with unremitting leg pain and progressive difficulty in bearing weight. Prior to this ED presentation, he was seen by his primary care physician and in a pediatric orthopedic clinic. Despite negative lower extremity radiographs and treatment with non-steroidal anti-inflammatory medications, his symptoms worsened, such that he refused to walk, with adaption of supine positioning with flexed lower extremities. There was no history of trauma, fever, systemic symptoms, or recent illness, and no change in his appetite, weight, stool, or urine. In the pediatric ED, the patient was afebrile with age-appropriate vital signs. On exam, he was lying in the supine position with his knees and hips flexed to his chest. He appeared uncomfortable and screamed with attempts to passively extend his hips or knees, though was noted to kick spontaneously. Palpation along his lower extremities revealed no focal tenderness, edema, warmth, or deformity. The remainder of his physical exam, including back, abdominal, and genitourinary, was otherwise unremarkable. He was given ketorolac for pain without significant improvement.

Initial labs revealed a white blood cell count of 17.6 with 81% neutrophils, C-reactive protein of 17.5 mg/L, erythrocyte sedimentation rate of 55 mm/hr, and normal hepatic function, LDH, uric acid, and creatine kinase. ED imaging included normal radiographs of the lumbar spine, pelvis, and lower extremities, normal abdominal ultrasound, and minimal left-sided joint effusion on hip ultrasound. The patient was admitted to the pediatric hospitalist service with pediatric orthopedic consultation and plan to obtain a sedated magnetic resonance imaging (MRI) of the pelvis, hips, and lumbar spine the following day.

## Hospital Course

**FIGURE 1:**
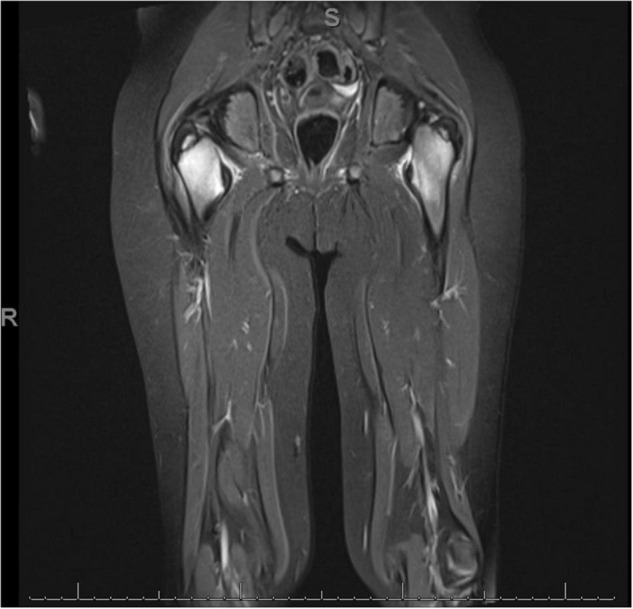
Magnetic resonance image of the patient’s bilateral lower extremities. Image demonstrates decreased T1 and increased T2 signal abnormality in the intertrochanteric femora bilaterally, right slightly greater than left.

Magnetic resonance imaging revealed bone marrow signal abnormality of the bilateral intertrochanteric femora with associated periosteal and adjacent soft tissue edema, along with marrow signal abnormality of the right proximal femoral diaphysis and bilateral distal femoral metaphyses and epiphyses (Figure 1). Radiology consultation supported a correlation with vitamin C deficiency and possibly chronic non-infectious osteomyelitis (CNO) or hematologic malignancy. Laboratory tests including vitamin C, celiac screen, cobalamin, fecal calprotectin, copper, and ceruloplasmin were sent. Pediatric gastroenterology, nutrition, and oncology services were consulted, and intravenous vitamin C was initiated.

Over the next few days, the patient continued to have pain and inability to bear weight. He gradually stopped eating due to worsening abdominal pain. On hospital day four, cobalamin resulted as >2000 pg/mL and his vitamin C level was normal, which correlated with his dietary history and did not support restrictive intake. While hypercobalaminemia can be associated with malignancy, the patient’s bone marrow biopsy revealed hypocellular marrow with intact lineages, suggesting an inflammatory rather than a hematologic malignancy (1). He did not undergo bone biopsy.

**FIGURE 2:**
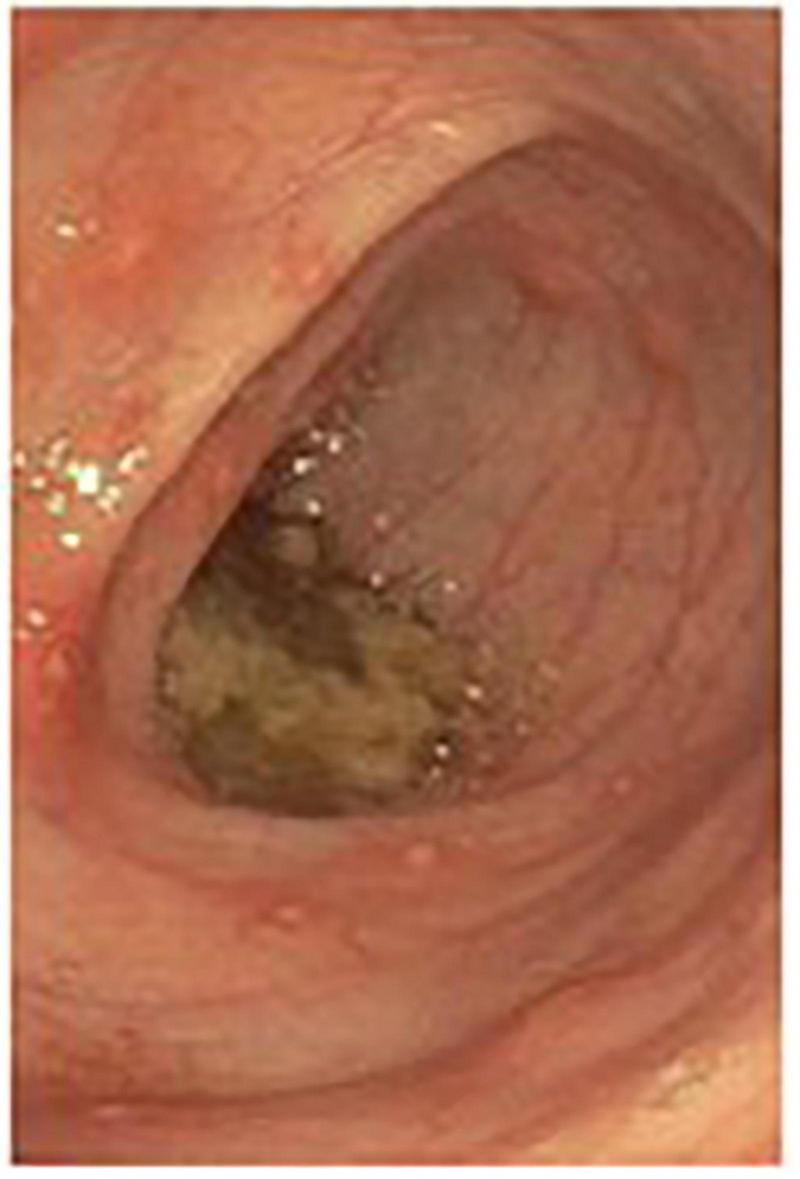
Erosions seen in the descending colon on colonoscopy.

Given the continued ambiguous clinical picture with persistent symptoms, a whole body MRI was obtained, revealing unchanged bone marrow signal abnormalities along with mild thickening, edema, and restricted diffusion of the ascending colon, suggesting a non-specific colitis. Parenteral nutrition was initiated and subsequently endoscopy and colonoscopy with biopsies were performed. Gross and microscopic examination revealed erythematous duodenopathy, erosive gastropathy, colonic erosions, anal fissure, hemorrhoids, multiple non-caseating granulomas, and diffuse colonic mucosal ulcerations consistent with Crohn’s disease (Figure 2).

Pulse methylprednisolone and Infliximab were administered, after which his oral intake and weight bearing ability significantly improved. He was subsequently discharged to a pediatric rehabilitation facility with gastroenterology follow-up and continued treatment with steroids and Infliximab. One year later, the patient remains on Infliximab and still without any gastrointestinal symptoms.

## Discussion

While limb pain and limp are common presentations in the pediatric ED, refusal to bear weight for more than a few days should prompt further investigation. The differential diagnosis includes traumatic injuries, infectious etiologies (e.g., osteomyelitis, septic arthritis), malignancy, inflammatory conditions (e.g., transient synovitis, acute myositis), rheumatologic and immunologic disorders (e.g., reactive arthritis, vasculitis), orthopedic complaints (e.g., Perthes disease, traction apophysitis), and abdominopelvic pathology (e.g., appendicitis, gonadal torsion).

Children with ASD are at greater risk for pathologic fractures secondary to potential nutritional deficiencies and low bone mineral density; whether they are at increased risk of non-accidental trauma remains controversial (2–4). In children with language delays, a careful physical examination may lead the clinician to a source of focal tenderness, thereby narrowing the diagnosis. When initial examination and imaging are non-revealing, cross sectional imaging may be helpful. In this patient, MRI findings of bone marrow signal abnormalities with periosteal reaction helped narrow the differential diagnosis to vitamin C deficiency, malignancy, and CNO.

Vitamin C is an essential nutrient required for collagen, neurotransmitter, and nitric oxide synthesis, as well as prostaglandin metabolism and fatty acid transport (5). Scurvy, the clinical syndrome resulting from vitamin C deficiency, can develop within 1–3 months of inadequate vitamin C intake. Non-specific complaints like fatigue, malaise, and loss of appetite may occur early in the course of disease, along with cutaneous symptoms (e.g., petechiae, bruising, perifollicular hemorrhage, and characteristic “corkscrew” hair), gingivitis, and poor wound healing (6). The majority of patients present with musculoskeletal manifestations like bone or joint pain, limp, and refusal to walk that may be severe enough to present as pseudoparalysis, particularly in infants and toddlers (7). The diagnosis of scurvy is clinical, though plasma and leukocyte vitamin C levels have fair reliability (8). MRI may reveal multifocal signal abnormalities, predominantly within the metaphyses, as well as bone marrow enhancement, periosteal elevation, and soft tissue signal abnormalities, as were seen in this patient’s imaging (9).

Scurvy is uncommon in the United States, and rarely occurs in children. Nonetheless, it has been reported among children with ASD and food selectivity (10–12). While there is a paucity of data on the prevalence of scurvy in children with ASD, a systematic review of twenty such case reports between 1990 and 2018 found the vast majority (85%) presented with refusal to walk (13). Prior case reports have also described scurvy as a mimicker of infectious osteomyelitis in children with ASD, though it has not previously been described as resembling non-infectious osteomyelitis (6, 14).

Malignancies that can present with bone marrow signal abnormality include acute lymphoblastic leukemia, acute promyelocytic leukemia, chronic myeloid leukemia, and infiltrative lymphoma (15, 16). Of these, B-cell acute lymphoblastic leukemia is most commonly associated with STIR bone marrow abnormality, and may occur prior to lymphoblast detection in the peripheral blood. Bone marrow biopsy may be necessary to exclude malignant infiltration as the underlying cause of clinical and radiographic findings.

Of the three conditions in our patient’s differential diagnosis, CNO is the rarest, with fewer than 500 cases reported in the literature worldwide (17). A minority of patients with CNO are found to have IBD. At the time of writing, there have been only 25 case reports of IBD-associated CNO among children and this is the first report in a child with ASD, for whom developmental/language delay and suspected nutritional deficiencies complicated the diagnosis (18–24). Among patients with IBD-associated CNO, it is common for CNO symptoms to precede gastrointestinal symptoms, which may also contribute to diagnostic delay (18). As with this patient, the majority of patients with IBD-associated CNO are ultimately found to have Crohn’s disease rather than ulcerative colitis (19).

While the etiology of CNO is unknown, it is described as a non-infectious, inflammatory disorder of the skeletal system, most commonly seen in children and adolescents. Most patients with CNO have a family history of autoimmune disorders, and it is hypothesized that CNO may involve the HLA-B27 pathway. However, due to lack of autoantibody and innate immune activation, it is typically described as an “autoinflammatory” rather than “autoimmune” disorder. Patients with CNO typically present with gradual onset of bone pain, local tenderness, and swelling. Areas of involvement can be symmetrical and frequently involve the metaphyses of long bones, clavicle, sternum, pelvis, and vertebrae, although lesions can occur anywhere in the skeleton (25). Non-specific systemic symptoms such as fever and malaise may be seen, but are uncommon.

Investigations may reveal mild leukocytosis and elevated inflammatory markers, as well as osteolytic and/or sclerotic bone lesions radiographically (21). MRI is particularly useful in the early stages of disease, before osteolysis and sclerosis can be detected on plain radiography. Typical MRI findings include the presence of bone marrow edema, osteolytic or sclerotic lesions, bone cortical thickening, and periosteal reaction (26–28). Cultures of bone lesions are sterile, and the diagnosis of CNO is typically based on history, physical exam, and radiological and histological findings, although it is considered a diagnosis of exclusion. In 2007, major and minor criteria were created to aid in the diagnosis of CNO (29).

Non-steroidal anti-inflammatory drugs are considered first-line treatment and have been shown to induce remission in a majority of patients (30). Other treatment options include corticosteroids, methotrexate, and sulfasalazine. In patients who fail first-line management, anti-tumor necrosis factor agents and bisphosphonates can be used (31, 32). CNO has a remission rate cited as anywhere from 40 to 80%, with characteristic intermittent periods of exacerbation and remission. Complications like limb-length asymmetries, kyphosis, and scoliosis may occur (33, 34). The patient presented here remains on Infliximab 12 months post diagnosis and is currently asymptomatic and in clinical remission with regards to both his Crohn’s disease and CNO.

## Conclusion

Chronic non-infectious osteomyelitis can be an elusive diagnosis that mimics scurvy, a nutritional deficiency that can affect children with ASD. Although rare, CNO can be the primary, presenting manifestation of pediatric IBD, particularly Crohn’s disease, and should be considered in the differential diagnosis of progressive failure to bear weight.

## Data Availability Statement

The original contributions presented in the study are included in the article/supplementary material, further inquiries can be directed to the corresponding author.

## Author Contributions

AG, BK, OA, and SB curated the data and wrote the manuscript. STS and GG-vS supervised and wrote the manuscript. All authors contributed to the article and approved the submitted version.

## Conflict of Interest

The authors declare that the research was conducted in the absence of any commercial or financial relationships that could be construed as a potential conflict of interest.

## Publisher’s Note

All claims expressed in this article are solely those of the authors and do not necessarily represent those of their affiliated organizations, or those of the publisher, the editors and the reviewers. Any product that may be evaluated in this article, or claim that may be made by its manufacturer, is not guaranteed or endorsed by the publisher.

## Abbreviations

CNO: chronic non-infectious osteomyelitis
IBD: inflammatory bowel disease
ASD: autism spectrum disorder
ED: emergency department
MRI: magnetic resonance imaging.
